# Vibratory Angioedema Due to Vigorous Nose Blowing: A Case Report

**DOI:** 10.7759/cureus.90197

**Published:** 2025-08-15

**Authors:** Tiffany Truong, Timothy Kaddis, Vivian Wang, Monica Tsai, Joseph Yusin

**Affiliations:** 1 Department of Internal Medicine, Cedars-Sinai Medical Center, Los Angeles, USA; 2 Division of Allergy and Immunology, Department of Internal Medicine, Veterans Affairs Greater Los Angeles Healthcare System, Los Angeles, USA; 3 Department of Medicine, David Geffen School of Medicine, University of California Los Angeles, Los Angeles, USA; 4 Division of Basic and Clinical Immunology, Department of Medicine, University of California Irvine School of Medicine, Orange, USA

**Keywords:** chronic inducible angioedema, chronic inducible urticaria, chronic rhino sinusitis, facial orolingual angioedema, vibratory angioedema

## Abstract

Vibratory angioedema is a rare type of chronic inducible angioedema with a variety of triggers reported in the literature. Standard management of vibratory angioedema involves avoidance of known triggers, as well as stepwise therapy starting with second-generation antihistamines. We present a unique case of vibratory angioedema provoked by vigorous nose blowing and improved with treatment of chronic rhinitis. To our knowledge, this is the first reported case of vibratory angioedema associated with this specific trigger. Recognition of nose blowing as a potential precipitant may have meaningful implications for diagnosis and management.

## Introduction

Urticaria is a dermatologic condition characterized by the presence of hives, angioedema, or both. Hives present as transient, well-circumscribed, pale, superficial skin lesions with a surrounding erythematous flare. They are typically pruritic and resolve within 24 hours. In contrast, angioedema manifests as deeper, skin-colored swelling, often with slower resolution and more associated discomfort compared to hives [1]. When symptoms persist for more than six weeks, the condition is classified as chronic urticaria [1]. Chronic inducible urticarias refer to forms consistently triggered by specific stimuli, such as cold, heat, or delayed pressure [1].

Mast cells play a central role in the pathophysiology of urticaria through degranulation and the release of histamine. Histamine, along with other pro-inflammatory cytokines, contributes to vasodilation and increased vascular permeability, leading to fluid leakage into the skin and surrounding tissues. However, the precise mechanisms by which physical stimuli trigger chronic inducible urticaria remain incompletely understood [2].

Taking a detailed and thorough clinical history is essential for diagnosis of chronic inducible urticarias, with emphasis on the timing of symptoms in relation to aggravating factors [3]. Provocation testing may be performed to confirm the diagnosis of chronic inducible urticaria and the procedure for each test depends on the trigger evaluated [3,4]. In the case of vibratory angioedema (VA), the provocation test involves applying a vortex mixer to the volar surface of the patient’s forearm for five to 10 minutes at a speed of approximately 1000 revolutions per minute, followed by monitoring the site for an additional 10 minutes [5]. A positive response is indicated by the development of localized urticaria, including edema, erythema, and pruritus at the site of stimulation.

Once the diagnosis is confirmed, initial management focuses on avoidance of known triggers. However, complete elimination of triggers may not always be realistic or feasible, and symptoms may persist, necessitating pharmacologic intervention [3,4]. First-line treatment consists of daily administration of standard-dose second-generation antihistamines. If symptoms continue, the dosage may be up-titrated to as much as four times the standard dose [3,4]. In patients who remain refractory to antihistamines, omalizumab is recommended at a dose of 300 mg every four weeks. If there is no clinical response after six months of therapy, cyclosporin may be considered as an alternative option [3,4].

VA is an uncommon subtype of chronic inducible urticaria provoked by vibratory stimuli. Common triggers include exercise, motor vehicle use, and showering [6]. Here we present a unique case of VA precipitated by vigorous nose blowing in a patient with chronic rhinosinusitis. This article was previously presented as a poster at the 2025 American Academy of Allergy, Asthma and Immunology/World Allergy Organization meeting on March 1, 2025.

## Case presentation

**Figure 1:**
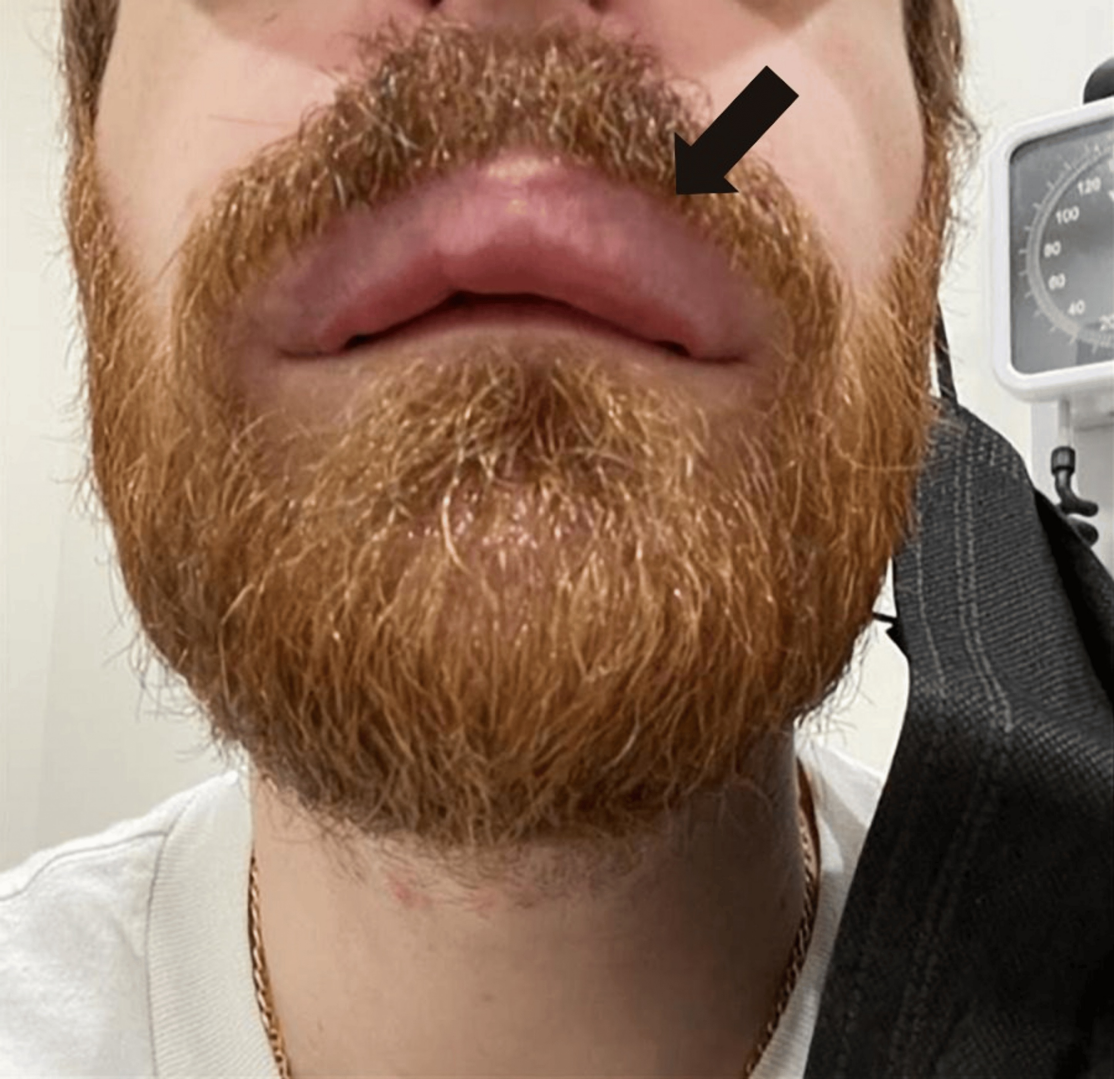
Upper lip angioedema.

A 36-year-old male with a history of nonallergic rhinitis presented to the allergy/immunology clinic for evaluation of five episodes of angioedema within the last 18 months. Each episode consisted of upper lip swelling, erythema, and numbness, which began within several minutes to 30 minutes after vigorous nose blowing. The swelling typically lasted several hours. Although these episodes were not observed in the clinic, the patient provided photographs documenting the upper lip swelling during prior events (Figure 1). He denied associated dyspnea, pruritus, rash, nausea, vomiting, diarrhea, fevers, chills, throat tightening, joint pain, or dysphagia. He reported no family history of similar episodes, chronic urticaria, or atopic or autoimmune conditions. He was not on any chronic medications at home.

The physical examination during this encounter was unremarkable, with no lip swelling, rash, or urticaria present. The patient’s detailed history, supported by photographic evidence of prior episodes, provided sufficient information to confirm the diagnosis. Given his symptoms were already well-documented, additional provocation testing could cause unnecessary discomfort without adding significant diagnostic value. For these reasons, we did not proceed with provocation testing.

Laboratory evaluation revealed normal tryptase, allergen environmental panel, C4, C1 esterase inhibitor level and function, C1q, complete blood count, and antinuclear antibodies (Table 1).

**Table 1:** Laboratory values. IgE, immunoglobulin E

Test	Units	Result	Reference range
Bermuda	k/uL	5.5	4.5-11.0
Hemoglobin	g/dL	13.9	13.3-17.7
Hematocrit	%	40.6	39-52
Platelets	k/uL	269.0	150-440
Eosinophils (%)	%	6.7	4.0-6.0
Eosinophil count	/uL	370.0	0-500
Tryptase	mcg/L	7.5	<11.0
C1Q complement protein	mg/dL	8.0	5.0-8.6
C1 inhibitor level	mg/dL	25.0	21-39
C1 inhibitor function	%	96.0	68-100
C4 level	mg/dL	26.0	15-53
Antinuclear antibodies screen		Negative	Negative
Olive tree IgE	kU/L	<0.10	<0.10
Walnut IgE	kU/L	<0.10	<0.10
Box elder IgE	kU/L	<0.10	<0.10
Maple leaf IgE	kU/L	<0.10	<0.10
Japanese cedar IgE	kU/L	<0.10	<0.10
Grey alder IgE	kU/L	<0.10	<0.10
Oak IgE	kU/L	<0.10	<0.10
Sycamore IgE	kU/L	<0.10	<0.10
Redtop bentgrass IgE	kU/L	<0.10	<0.10
Burmuda grass IgE	kU/L	<0.10	<0.10
Brome grass IgE	kU/L	<0.10	<0.10
Lambs quarter IgE	kU/L	<0.10	<0.10
Russian thistle IgE	kU/L	<0.10	<0.10
Mugwort IgE	kU/L	<0.10	<0.10
Pigweed IgE	kU/L	<0.10	<0.10
Ragweed IgE	kU/L	<0.10	<0.10
False ragweed IgE	kU/L	<0.10	<0.10
Oat pollen IgE	kU/L	<0.10	<0.10
Dermatophagoides pteronyssinus IgE	kU/L	<0.10	<0.10
Dermatophagoides farinae IgE	kU/L	<0.10	<0.10
Cladosporium IgE	kU/L	<0.10	<0.10
Aspergillus fumigatus IgE	kU/L	<0.10	<0.10
Dog dander IgE	kU/L	<0.10	<0.10
Cat dander IgE	kU/L	<0.10	<0.10
Cockroach IgE	kU/L	<0.10	<0.10

The patient was started on intranasal fluticasone and azelastine for rhinitis and, at seven-month follow-up, reported a significant reduction in symptoms of both rhinitis and angioedema.

## Discussion

The differential diagnosis included acquired angioedema due to C1-inhibitor deficiency or hereditary angioedema; however, normal C4, C1 esterase inhibitor level and function, and C1q made these unlikely. There was no recent use of an angiotensin converting enzyme (ACE) inhibitor to suggest ACE-inhibitor-induced angioedema. A normal tryptase level did not support the diagnosis of an underlying mast cell disorder. Furthermore, autoimmune etiologies such as vasculitis were considered unlikely given the negative review of systems and absence of autoantibodies.

Among the various types of chronic inducible urticarias (cold, heat, delayed pressure, solar, dermatographia, vibratory, cholinergic, and aquagenic urticaria), vibratory and delayed pressure urticaria were strongly considered. Delayed pressure urticaria was deemed unlikely given the consistent early onset of symptoms immediately following nose blowing. Thus, vibration was identified as the most likely physical trigger. We propose that elevated intranasal pressure generated during nose blowing was transmitted to the soft tissues of the lips as a vibratory stimulus, leading to symptom onset.

In light of the patient’s history and concurrent improvement in both rhinitis and angioedema with treatment of his rhinitis, we diagnosed the patient with vibratory angioedema induced by forceful nose blowing. VA can be caused by a variety of physical activities, and the mainstay of treatment is trigger avoidance when possible. Frequently reported triggers include exercise, massage, showering, toweling, and using motor vehicles [6]. Infrequently reported precipitants of VA include playing musical instruments, snoring, and sexual intercourse [6]. To our knowledge, this is the first published case in the English literature to describe nose blowing as a distinct cause of VA. Given the unusual nature of this trigger, it is likely underrecognized, and increased awareness may help expedite diagnosis and guide effective management to reduce episodes of recurrence. Early diagnosis through thorough history taking also spared the patient from more costly and burdensome therapies.

International guidelines for the treatment of chronic urticaria recommend a stepwise approach beginning with a second-generation antihistamine, which can be up-titrated to four times the standard dosing [4,7]. For refractory cases to antihistamines, omalizumab is second-line therapy, followed by cyclosporine as third-line therapy [4,7]. Alternatively, our patient achieved a significant reduction in symptoms with standard therapy for rhinitis alone. This case highlights the clinical significance of nose blowing as a potential trigger of VA, demonstrating that targeted management of the underlying condition can improve treatment efficiency and prevent unnecessary pharmacologic escalation. Similarly, a rare case of VA precipitated by snoring was reported in the literature. The patient demonstrated a complete resolution of VA symptoms with subsequent treatment of sleep apnea with continuous positive airway pressure [8].

## Conclusions

Vibratory angioedema is an uncommon subtype of chronic inducible angioedema with diverse potential triggers. Thorough history taking is essential for accurate diagnosis and identification of precipitating factors. Increased awareness of nose blowing as a distinct trigger of vibratory angioedema is warranted, as its recognition facilitates timely diagnosis and targeted management of the underlying condition, thereby sparing patients from unnecessary testing and escalation of treatments. In our patient, targeted treatment of chronic rhinitis prompted marked improvement of symptoms related to vibratory angioedema.
